# Long-Term Changes in Parameters of Bone Quality in Kidney Transplant Recipients Treated with Denosumab

**DOI:** 10.1007/s00223-025-01349-x

**Published:** 2025-02-21

**Authors:** Francesco Pollastri, Angelo Fassio, Pietro Manuel Ferraro, Stefano Andreola, Giovanni Gambaro, Andrea Spasiano, Chiara Caletti, Lisa Stefani, Matteo Gatti, Paolo Fabbrini, Maurizio Rossini, Isotta Galvagni, Davide Gatti, Giovanni Adami, Ombretta Viapiana

**Affiliations:** 1 Rheumatology Unit, University of Verona, Policlinico GB Rossi, 37134 Verona, Italy; 2 Nephrology Unit, University of Verona, Verona, Italy; 3 Department of Nephrology and Dialysis, Ospedale Bassini, ASST Nord Milano-Cinisello Balsamo, Milan, Italy

**Keywords:** Kidney transplant recipients, Denosumab, Indices of bone quality, DXA, TBS, 3D-DXA

## Abstract

Kidney transplant recipients (KTRs) have an elevated fracture risk. While dual-energy X-ray absorptiometry (DXA) is commonly used to assess areal bone mineral density (aBMD), it does not capture all aspects of bone quality. We investigated the long-term effects on bone DXA-derived indices of bone quality in KTRs treated with denosumab and untreated with denosumab. This is a retrospective study, including KTRs treated with denosumab and untreated age and sex-matched KTR controls. DXA-derived parameters, including trabecular bone score (TBS) and 3D-DXA parameters, were measured at the lumbar spine and femur at baseline and after four years. Hierarchical linear models were used to assess the between-group effect of treatment over time, also adjusting for site-specific aBMDs. We enrolled 23 KTRs treated with denosumab and 23 KTR denosumab-untreated KTRs. Significant between-group differences over time in favor of the denosumab group were observed for TBS (0.843, 95%CI 0.439; 1.248,*p* < 0.001), trabecular volumetric BMD at the total hip (Tb.vBMD TH) (13.492, 95%CI 1.707; 25.278, *p* = 0.003), cortical volumetric BMD at the femoral neck (Ct.vBMD FN) (28.766, 95%CI 8.373; 49.158, *p* = 0.008), cortical surface BMD at the total hip (c.sBMD TH) (10.507, 95%CI 4.140; 16.873,*p* = 0.002), cortical surface at the femoral neck (c.sBMD FN) (8.795, 95%CI 2.818; 14.771, *p* = 0.006), and cortical thickness at the total hip (Ct.th.TH) (0.075, 95%CI 0.020; 0.130, *p* = 0.010). After adjusting for BMD, the differences on TBS and Ct.vBMD FN and c.sBMD FN remained significant. Denosumab treatment in KTRs was associated with better outcomes in terms of bone quality and geometry parameters, independent of changes in aBMD.

**Supplementary Information:**

The online version contains supplementary material available at 10.1007/s00223-025-01349-x.

## Introduction

Chronic kidney disease (CKD) represents an increasing global health issue with an age-standardized prevalence of approximately 9% in high-income countries [1]. Even at mild stages of severity, CKD is associated with disturbances in calcium–phosphorus metabolism known as CKD-Mineral and Bone Disorder (CKD-MBD) [2, 3]. The consequent rise in fracture risk of CKD-MBD patients also affects kidney transplant recipients (KTR), who exhibit a fracture prevalence of around 35% with a high impact on morbidity and mortality [4, 5].

A growing body of evidence supports the ability of Dual-energy X-ray Absorptiometry (DXA) to predict fracture risk in patients with CKD, including KTR [6–8]. However, the measurement of areal bone mineral density (aBMD) does not identify all fragility fractures in the general population [9, 10]. Given that DXA-measured aBMD explains 60–70% of the variability in bone strength, there are key elements that are not captured by it, and other determinants such as bone geometry and trabecular microarchitecture also need to be considered. Therefore, assessing indices estimating bone microarchitecture and geometry/structure may offer a more comprehensive insight into how tissue quality contributes to overall bone strength [11].

These considerations might be even more impactful after kidney transplantation, where the measure of bone mass doses not predict the type of osteodistrophic lesion and may lead to inaccurate conclusions [12].

In recent years, several bone quality indices have been developed to evaluate bone microarchitecture, enhancing fracture risk assessment beyond aBMD alone [13]. Some of these parameters, such as the Trabecular Bone Score (TBS), have been investigated in the population of patients with CKD in whom assessing bone quality is particularly important due to the frequent disturbances in bone metabolism [14–19].

The 3D bone shape and density distribution of the proximal femur can be analyzed from 2D-DXA scans using 3D modeling. This technique, referred to as 3D-DXA, uses a statistical model based on a database of quantitative computed tomography (QCT) scans to generate a 3D patient-specific model of the proximal femur that allows for a separate characterization of the cortical and trabecular bone compartments. The accuracy of 3D-DXA parameters and measurements was evaluated against same patient quantitative computed tomography parameters [20, 21].

For KTRs with low BMD, the first year post-transplant treatments typically include vitamin D, antiresorptive agents, or a combination of both [2]. Denosumab, a monoclonal antibody directed toward the receptor activator of nuclear factor kappa-B, has been shown to be effective for bone loss and improving bone quality, including in long-term therapies in the general population [22–24]. Recent research confirms its safety and efficacy in KTRs in the early period after transplantation [25]. Recently published data from this cohort confirmed a benefit in terms of BMD even in patients who began denosumab treatment several years after transplantation [26].

Currently, data on both the changes of TBS and 3D-Shaper in KTRs subjects treated with denosumab are completely lacking. Therefore, we performed a pre-post-study with a nonequivalent control group to investigate the long-term (four years) changes in DXA-derived measurements of estimated bone quality in KTRs receiving treatment with denosumab, compared to a cohort of age- and sex-matched densumab-untreated KTRs. In addition, we explored the unique changes of these indices, both in the treatment and in the control groups, independent of changes in BMD.

## Materials and Methods

### Study Design

This retrospective study with nonequivalent control group study aimed to evaluate the impact of denosumab treatment on various bone parameters derived from DXA in patients with osteoporosis.

We reviewed all KTRs who, upon physician’s decision (based on the clinician’s estimation of fracture risk), started treatment with denosumab 60 mg every six months from January 2014 to January 2018. Patients who received continuous treatment for at least four years, with available baseline and four-year follow-up DXA scans were included.

An untreated cohort was selected among the overall pool of KTRs referring to the clinic who had available baseline and four-year follow-up DXA scans. All KTRs of the denosumab group were then individually age and sex-matched with untreated KTRs with a 1:1 ratio (± 3-year tolerance for age). In the case of two or more potential eligible matches, the closest one in terms of age was selected.

The study therefore included two groups: KTRs treated with denosumab and a control group of age- and sex-matched KTRs not receiving denosumab. Participants were assessed at baseline and during follow-up using DXA to analyze aBMD at the lumbar spine (LS), total hip (TH), and femoral neck (FN). The full details of the protocol, inclusion/exclusion criteria, and the main results including the aBMD data have been published previously [26].

In brief, the study was conducted at the Joint Rheumatology and Nephrology bone clinic, University of Verona (Verona, Italy).

### Measured Parameters

Ten DXA-derived bone were assessed.

TBS T-scores were obtained at the LS at baseline and follow-up for both groups (GE TBS Insight 3.0.3.0). TBS is a texture index that evaluates pixel gray-level variations in the lumbar spine DXA images, providing an indirect measure of bone microarchitecture [27].

3D-DXA analysis at the proximal femur were performed at baseline and follow-up for both groups by 3D-Shaper software v2.11 (3D-Shaper Medical, Barcelona, Spain) as previously described with operators blinded to treatment [21]. Cortical volumetric BMD at Total Hip (Ct.vBMD TH), Trabecular vBMD at Total Hip (Tb.vBMD TH), Cortical vBMD at Femoral Neck (Ct.vBMD FN), Trabecular vBMD at Femoral Neck (Tb.vBMD FN), Cortical surface BMD at Total Hip (c.sBMD TH), Cortical surface BMD at Femoral Neck (c.sBMD FN), Cortical thickness at the Total Hip (Ct.th TH), Cortical thickness at the Femoral Neck (Ct.th FN), and Cross-Sectional Moment of Inertia (CSMI) – Intertrochanteric. were therefore estimated [20, 21].

### Statistical Analysis

Student’s *t* test for independent samples was used to test differences between the two groups and for normally distributed variables, Mann–Whitney *U* test for non-normally distributed variables, and the chi-square test was used to compare proportions. Paired-samples *t* tests for each group were conducted to analyze changes versus baseline. To estimate the effect size (ES) of each parameter change for each group, Cohen’s d were also calculated, and interpreted as 0.2 = small effect 0.5 = moderate effect, and 0.8 = large effect [28].

Correlation between each bone parameter and the respective site-specific BMD was tested through Pearson’s correlation. The strength of correlation, expressed as r values, were interpreted as usual: 0.00–0.10 negligible correlation, 0.10–0.39 weak correlation, 0.40–0.69 moderate correlation, 0.70–0.89 strong correlation, and 0.90–1.00 very strong correlation [29].

To assess the effect of denosumab treatment over time and to compare the between-group changes over time of the selected bone parameters, hierarchical linear models were employed. HLMs were chosen due to their ability to handle longitudinal data with repeated measurements, accounting for both inter-individual and intra-individual variability.

For each bone parameter, two distinct nested HLMs were constructed.

1) The reduced model: for each parameter, a model was developed to test the interaction between time (baseline vs. follow-up) and treatment group (denosumab-treated vs. untreated). This model was used to evaluate whether denosumab treatment influenced changes in the bone parameter over time compared to the control group. The general structure of this model was as follows: Parameter∼Time × Group + (1∣Subject ID).

2) The full model: each primary model was further adjusted for the corresponding BMD value at the site of measurement (e.g., total hip aBMD for cortical total hip vBMD). This adjustment was introduced to isolate the unique effect of denosumab on the parameter of interest, independent of BMD changes at the site of observation. The adjusted model accounted for the specific BMD value to control for its potential confounding effect: Parameter∼Site-Specific BMD + Time × Group + (1∣Subject ID).

In both models, patients were clustered by subject (‘Subject ID’) to account for the repeated measures design and to control for intra-individual variability over time. This approach allowed for a more nuanced understanding of the direct impact of denosumab on bone parameters, distinguishing it from changes attributable to corresponding BMD variations, ensuring a more robust evaluation of denosumab’s efficacy on the studied bone parameters, considering both the time-dependent interaction and the influence of site-specific BMD.

Finally, to investigate the potential predictors of aBMD changes over time (expressed as percentage changes from baseline at the lumbar spine, total hip and femoral neck), a Random Forest (RF) model was constructed using the ‘cforest’ function from the party package in R to handle all the predictor variables (measured at baseline) in an unbiased and robust to overfitting manner [30]. The dataset included the following twenty-six variables as predictors: treatment, gender, BMI, presence of type 2 Diabetes Mellitus, concurrent treatment with calcitriol, concurrent treatment with calcitriol cinacalcet, history of fractures, history of pre-transplant dialysis (yes/no), creatinine, ALP, PTH, 25-hydrody vitamin D, serum calcium concentrations (corrected), TBS T-Score LS, Ct.vBMD TH, Tb.vBMD TH, Ct.vBMD FN, Tb.vBMD FN, CSMI intertroch., aBMD LS, aBMD TH, aBMD FN, c.sBMD TH, c.sBMD FN, Ct.th TH, and Ct.th FN.

The RF model classified the predictors based on their importance (measured as the mean decrease in accuracy when permuted). As illustrated by Fife et al. [30], the top three variables were then selected for further analysis with a linear regression model.

All statistical analyses were performed using R-studio (version 2024.04.0), with hierarchical linear models executed using the ‘lme4’ and ‘lmerTest,’ ‘flexplot’ and ‘party’ packages. Statistical significance was set at *p* < 0.05.

## Results

The CONSORT flowchart relative to the present study is reported in Supplementary Fig. 1. Of the four excluded individuals of the denosumab group, three were excluded because of incomplete DXA data and one due to the loss in follow-up (moved to a different healthcare facility). The timeframe separating the baseline and follow-up DXA scan was (mean, standard deviation) 49 (3) months (range 42–55 months) for the denosumab group and 49 (4) months (range 41–55 months) for the untreated group (F(1, 44) = 0.14, *p* = 0.71 between groups).

The baseline characteristics of the two groups are reported in Table 1; the distribution of the baseline CKD stages is depicted in Supplementary Fig. 2; none of the patients enrolled presented an eGFR < 30 mL/min/1.73 m2. All patients were in stable treatment with a calcineurin inhibitor, mycophenolic acid, and glucocorticoids (5 mg daily of prednisolone equivalent). As expected, the baseline BMD and T-scores at all three sites were significantly lower in the denosumab group, while no significant differences were observed for the other parameters.

**Table 1:** Baseline characteristics of the two groups

	Denosumab (N = 23)	Controls (N = 23)	*p* value
Sex (M:F)	10:13	10:13	Ns
Age (years)	61.50 (7.20)	61.40 (9.00)	Ns
BMI (kg/m^2^)	23.10 (1.80)	24.30 (4.30)	Ns
TBS T-score	− 2.59 (1.20)	− 1.46 (1.23)	0.003
T2DM	3 (15%)	3 (15%)	Ns
History of previous haemodialysis	14 (61%)	17 (74%)	Ns
Years from transplant to baseline DXA; median [IQR]	4 [1.5;10]	5 [2;11]	Ns
Chronic GCs	23 (100%)	23 (100%)	/
Treatment with calcimimetics	4 (17%)	6 (26%)	Ns
Treatment with active vitamin D compounds	6 (26%)	7 (30%)	Ns
25OHD (nmol/L)	48.6 (26.7)	57.2 (37.3)	0.067
ALP (U/L)	83.4 (43.5)	77.8 (29.1)	Ns
PTH (pmol/L)	13.0 (10.1)	18.9 (14.8)	Ns
Serum creatinine (mg/dL)	1.31 (0.45)	1.27 (0.43)	Ns
LS T-score	− 2.70 (1.20)	− 1.42 (1.70)	0.006
TH T-score	− 2.35 (0.77)	− 1.40 (1.16)	0.014
FN T-score	− 2.58 (0.52)	− 1.98 (1.04)	0.002
Cortical vBMD TH (mg/cm3)	678.00 (81.30)	734.00 (95.70)	0.038
Trabecular vBMD TH (mg/cm3)	99.70 (37.90)	122.00 (39.40)	Ns
Cortical vBMD FN (mg/cm3)	701.00 (69.20)	746.00 (85.00)	Ns
Trabecular vBMD FN (mg/cm3)	149.00 (36.50)	166.00 (43.80)	Ns
c.sBMD TH	119 (22.3)	133 (28.0)	Ns
c.sBMD FN	106 (18.1)	111 (21.3)	Ns
Ct.th.TH	1.75 (0.14)	1.79 (1.19)	Ns
Ct.th.FN	1.57 (0.17)	1.53 (1.18)	Ns
CSMI intertrochanteric (cm4)	5.07 (2.10)	5.35 (2.87)	Ns

Data are expressed as mean (SD) or absolute numbers. 25OHD, 25-hytrody vitamin D serum levels; *ALP* Alkaline phosphatase, *BMI* Bone mass index, *CSMI* Cross-sectional moment of inertia, *FN* Femoral neck, *GCs* Glucocorticoids, *PTH* Parathyroid hormone, *TBS* Trabecular bone score, *TH* Total hip, *vBMD* Volumetric bone mineral density, *c.sBMD* Cortical surface BMD, *Ct.th* Cortical thickness

The main results including aBMD data have already been published previously [26]. Briefly, the denosumab group showed a significant increase from baseline in aBMD at the LS (9.0 ± 10.7%, *p* < 0.001) and TH (3.8 ± 7.9%, *p* = 0.041). The untreated group showed a significant decrease at all sites (− 3.0 ± 7%, *p* = 0.041 at the LS; − 6.3 ± 9.2%, *p* = 0.003 at the TH; − 6.7 ± 9.3%, *p* = 0.003 at the FN). The between-group differences in percent BMD changes were statistically significant at all sites.

**Fig. 1:**
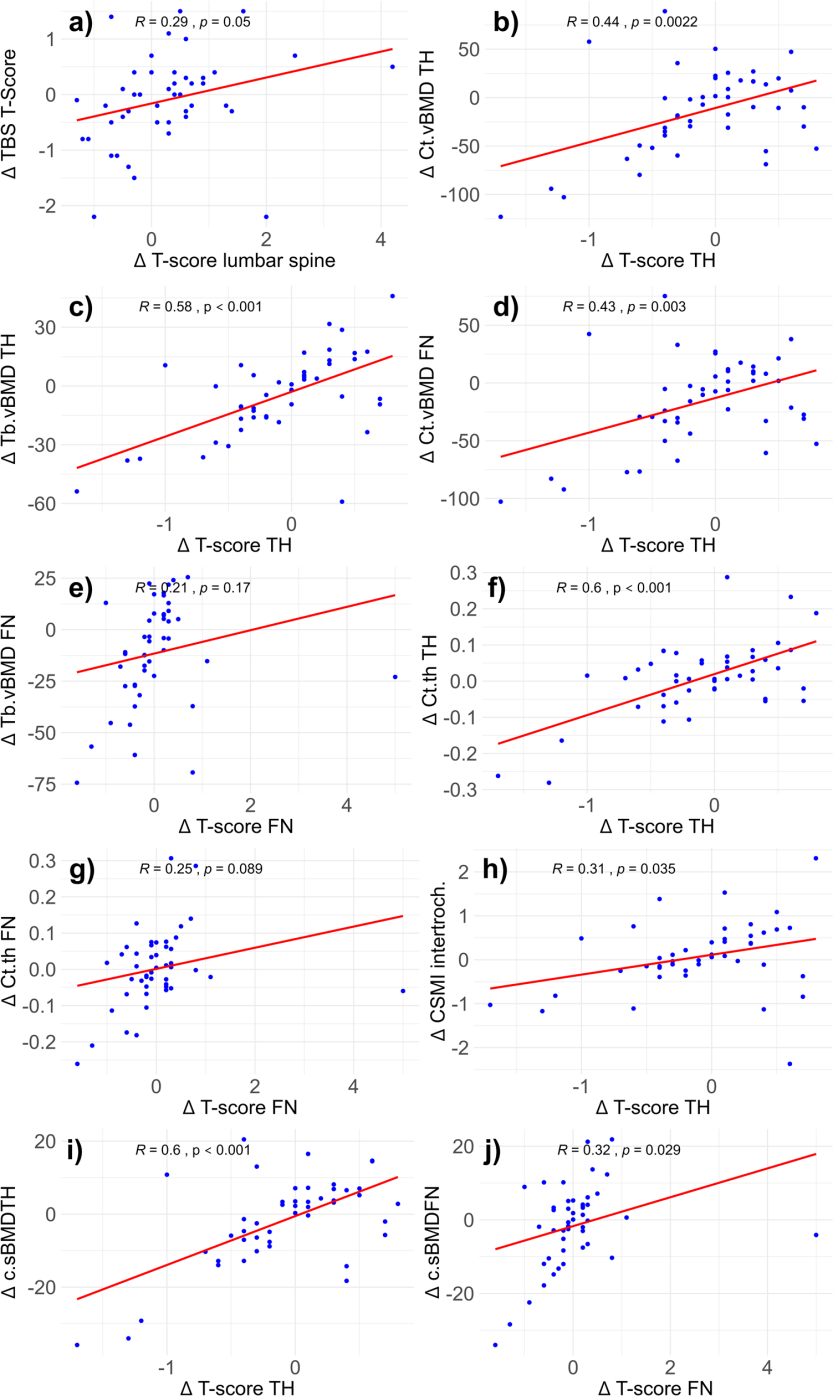
Bivariate correlations between the investigated indices of estimated bone quality and their respective site-specific BMD

**Fig. 2:**
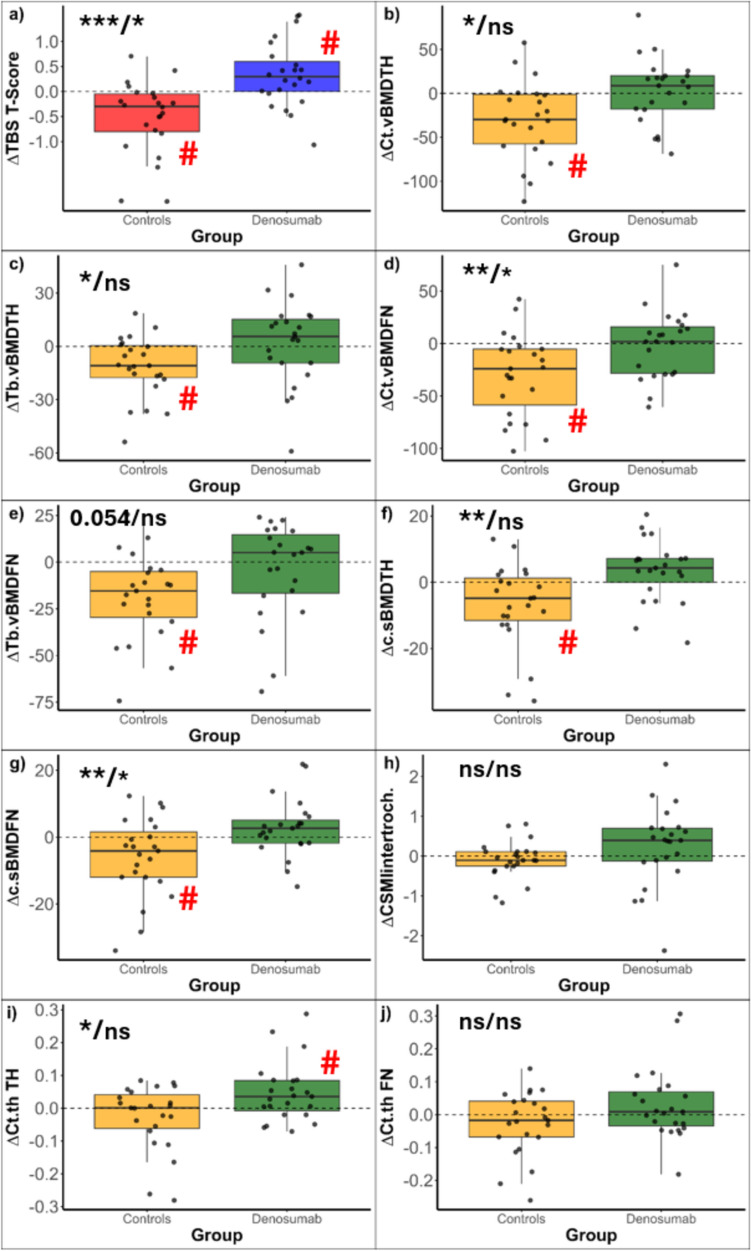
Boxplot depicting the absolute changes (∆) of the TBS T-Score of the other indices over time in the treated and untreated groups. Time*group interaction p value (crude/adjusted for ∆BMD): * *p* < 0.05, **p < 0.01, *** *p* < 0.001, and # *p* < 0.005 vs baseline. CSMI, cross-sectional moment of inertia; c.sBMD, cortical surface BMD; Ct.vBMD, cortical volumetric bone mineral density; Ct.th, cortical thickness; FN, femoral neck; Tb.vBMD, trabecular vBMD; TBS, trabecular bone score; TH, total hip

The correlations between the pre-post (four-year) changes of each parameter and the changes at the site-specific BMD are reported in Fig. 1. The absolute changes of the TBS T-Score of the other indices over time in the treated and untreated groups are reported in Fig. 2. Treatment with denosumab was associated with statistically significant improvements of the TBS (Cohen’s d 0.5, moderate ES, *p* = 0.02 vs. baseline), and Ct.th TH (Cohen’s d 0.52, moderate ES, *p* = 0.026 vs baseline).

The control group was associated with statistically significant worsening of the TBS (Cohen’s d -0.7, moderate ES, *p* = 0.003 vs baseline), Ct.vBMD TH (Cohen’s d -0.68, moderate ES, *p* = 0.004 vs baseline), Tb.vBMD TH (Cohen’s d-0.68, moderate ES, *p* = 0.004 vs baseline), Ct.vBMD FN (Cohen’s d − 0.77, moderate ES, *p* = 0.001 vs baseline), Tb.vBMD FN (Cohen’s d -0.81, large ES, *p* < 0.001 vs baseline), c.sBMD TH (Cohen’s -0.55, moderate ES, *p* = 0.014 vs baseline), and c.sBMD FN (Cohen’s -0.51, moderate ES, *p* = 0.024).

A statistically significant difference in the parameters changes between the denosumab and control group (time*group interaction) was observed for seven out of ten of the selected parameters, namely TBS, Ct.vBMD TH, Tb.vBMD TH, Ct.vBMD FN, c.sBMD TH, c.sBMD FN, and Ct.th TH (Fig. 2, Table 2). After adjustment for the respective site-specific BMD (full model data, Table 3), the changes of TBS and Ct.vBMD FN and c.sBMD FN remained statistically significant; after adjustment for the FN BMD, treatment with denosumab versus no treatment was estimated to be associated with higher values of TBS T-score of 0.569 (*p* = 0.016) and of FN Ct.vBMD of 24.6 mg/cm3 (*p* = 0.024) and c.sBMD FN of 7.1 mg/cm2 (*p* = 0.025).

**Table 2:** Reduced model for each site. The model reports the estimates for the time*group interaction and their 95%CI. 95%CI, 95% confidence interval; CSMI, cross-sectional moment of inertia; vBMD, volumetric bone mineral density

TBS T-score: reduced model			
Predictor	estimate	95%CI	*p* value
Time*group	0.843	0.439; 1.248	< 0.001
**Cortical total hip vBMD (mg/cm3): reduced model**			
Time*group	32.251	8.735; 55.767	0.010
**Trabecular total hip vBMD (mg/cm3): reduced model**			
Time*group	13.492	1.707; 25.278	0.030
**Cortical femoral neck vBMD (mg/cm3): reduced model**			
Time*group	28.766	8.373; 49.158	0.008
**Trabecular femoral neck vBMD (mg/cm3): reduced model**			
Time*group	14.180	0.117; 28.241	0.055
**Cortical surface BMD total hip (mg/cm**^**2**^**): reduced model**			
Time*group	10.507	4.140; 16.873	0.002
**Cortical surface BMD femoral neck (mg/cm**^**2**^**): reduced model**			
Time*group	8.795	2.818; 14.771	0.006
**Cortical thickness total hip (mm): reduced model**			
Time*group	0.075	0.020; 0.130	0.010
**Cortical thickness femoral neck (mm): reduced model**			
Time*group	0.056	− 0.002; 0.116	0.067
**CSMI intertroch (cm4): reduced model**			
Time*group	0.338	− 0.108; 0.785	0.145

**Table 3:** full model (site-specific BMD adjusted) for each site

TBS T-score: full model			
Predictor	estimate	95%CI	*p* value
LS T-score	0.290	0.138; 0.439	< 0.001
Time*group	0.569	0.116; 1.008	0.016
**Cortical total hip vBMD (mg/cm3): full model**			
TH T-score	53.613	37.188; 69.570	< 0.001
Time*group	− 0.149	− 26.018;24.816	0.991
**Trabecular total hip vBMD (mg/cm3): full model**			
LS T-score	26.023	19.365; 32.641	< 0.001
Time*group	− 2.235	− 13.168; 8.610	0.692
**Cortical femoral neck vBMD (mg/cm3): full model**			
FN T-score	13.549	3.227; 24.312	0.012
Time*group	24.642	4.020; 45.040	0.024
**Trabecular femoral neck vBMD (mg/cm3): full model**			
LS T-score	14.612	7.094; 22.500	< 0.001
Time*group	9.732	− 5.584; 24.621	0.215
**Cortical surface BMD total hip (mg/cm**^**2**^**): full model**			
TH T-score	17.027	12.769; 21.212	< 0.001
Time*group	0.216	− 6.204; 6.435	0.947
**Cortical surface BMD femoral neck (mg/cm**^**2**^**): full model**			
LS T-score	5.536	2.585; 8.633	< 0.001
Time*group	7.110	1.124; 13.006	0.024
**Cortical thickness total hip (mm): full model**			
LS T-score	0.111	0.079; 0.143	< 0.001
Time*group	0.008	− 0.042; 0.059	0.749
**Cortical thickness femoral neck (mm): full model**			
FN T-score	0.043	0.015; 0.072	< 0.001
Time*group	0.043	− 0.016; 0.102	0.947
**CSMI intertroch (cm4): full model**			
TH T-score	1.020	− 0.059; 0.690	< 0.001
Time*group	− 0.278	− 0.838; 0.245	0.307

The model reports the estimates for LS T-score and for the time*group interaction and their 95%CI. 95%CI, 95% confidence interval; *CSMI* Cross-sectional moment of inertia, *vBMD* Volumetric bone mineral density, *CSMI* Cross-sectional moment of inertia, *vBMD* Volumetric bone mineral density

The variable importance of the three RF models for predicting percent aBMD changes over time at the LS, TH, and FN is reported in Supplementary Table 1. Linear regression models for the prediction of percentage aBMD changes at the LS, TH and FN including the variables selected with the RF algorithm are reported in Supplementary Table 2. Of all the selected variables, only treatment with denosumab vs. no treatment resulted to be a statistically significant predictor for percentage aBMD changes over time at all the three sites. In addition, limitedly at the LS site, higher baseline PTH values were negatively associated with percentage BMD changes (estimate: − 0.23, *p* = 0.048), while BMI was positively associated (estimate: 1.52, *p* = 0.00129).

## Discussion

This study investigated the application of bone quality indices derived from DXA in KTRs treated with denosumab compared to untreated controls. Our findings demonstrate a beneficial effect of denosumab on two bone quality indices: TBS and cortical thickness at the total hip. The improvements in the denosumab group were small to moderate in effect size, while the untreated control group showed a significant decline in bone quality. The deterioration in the control group, particularly in TBS and six of the nine 3D-Shaper indices, highlights the potential value of denosumab not only in improving bone quantity but also in potentially preserving bone quality over time.

The significant positive effects of denosumab on bone parameters, such as TBS and CSMI in the intertrochanteric and lower shaft regions, are consistent with its known effects on BMD. Interestingly, despite the stabilization or improvement of most parameters in the denosumab group, the untreated group showed a marked decline at several sites. Moreover, the ES of this disease in the control group was more pronounced than the benefit observed in the treatment group. This observation suggests that the bone health impairment in the absence of treatment might be quicker and of greater magnitude than the gains associated with denosumab. Thus, the importance of a regularly scheduled follow-up in these high-risk subjects.

The use of HLMs incorporating changes in aBMD allowed us to conclude that the associations found for denosumab treatment might be at least in part independent of BMD changes, with significant differences persisting for TBS and Ct.vBMD at the femoral neck after adjusting for site-specific BMD. This suggests that denosumab might provide additional benefits in preserving bone quality.

Our findings align with prior research, including the sub-analysis of FREEDOM trial involving subjects with CKD [25], and our previous aBMD data on this same real-life sample [26].

Indeed, in the denosumab-untreated group, we observed a striking worsening at both the LS (TBS) and at the femur, especially at the trabecular and cortical vBMD of the FN. Once again, we emphasize that, in the absence of treatment, KTRs subjects seem to be destined to a significant widespread deterioration not only measures of bone density but also microarchitecture and geometry. On the other hand, treatment with denosumab is associated with stabilization or even improvement at certain compartments (including cortical thickness at the FN). In addition, these findings highlight the importance of assessing both cortical and trabecular components of bone when evaluating bone health, particularly in KTRs with complex bone metabolism. Interestingly, in a bone histomorphometry study on iliac biopsies from the FREEDOM trial, when analyzing the degree of bone mineralization (DMB), the authors found significant increases after two to three years of denosumab treatment for both trabecular and cortical bone; however, the increase was more pronounced in cortical bone [31]. Indeed, after two to three years, the median trabecular bone DMB was still below that of the premenopausal reference group and only exceeded it at year five, while the cortical DBM well exceeded it at the 2/3-year mark. Therefore, we might speculate that in a site where both cortical and trabecular bone are well represented, more than five years of treatment might be needed to consistently demonstrate increases in all its components.

The results of this study also suggest the potential utility of DXA-derived bone quality parameters, such as TBS and 3D-DXA parameters, in monitoring bone health in KTRs. The observed increase in TBS T-scores following denosumab treatment suggests that TBS may provide valuable insights into changes in bone microarchitecture, complementing traditional aBMD measurements. Notably, after adjusting for aBMD changes, denosumab was associated with a positive difference of approximately 0.57 points in the TBS T-score in favor of the treated group, suggesting a benefit that extends beyond aBMD improvements alone; the femur showed a similar pattern especially at the cortical compartment of the FN, which was however mainly driven by the significant worsening within the denosumab-untreated group (Ct.vBMD and c.sBMD). This suggests the intriguing challenge of differentiating the treatment effects at the different femoral compartments, which may, as already mentioned, respond with different timings and magnitudes. It is important to note, however, that in the absence of adequately powered studies, we do not yet know whether the increase in TBS or 3D-DXA parameters are indeed associated with fracture risk reduction in KTRs.

Denosumab has been shown to be effective in improving hip structural parameters in non-nephropathic patients with osteoporosis and in subjects with CKD and haemodialysis [32–38]. Our data confirm these findings in KTRs as well, suggesting an effect on trabecular and cortical bone also in this special population. Indeed, our data are also in line with a microarchitecture ancillary study with high-resolution peripheral QCT (radius and tibia) by Bonani et al. [39]. A particularly striking observation is that the absence of treatment is associated with significant declines in most of the investigated indices, further emphasizing the importance of strict follow-up in these patients given the high risk of developing significant bone fragility over time.

Although BMD remains a crucial tool in fracture risk assessment, especially in patients with CKD, its limitations are increasingly recognized, particularly in KTRs. Bone disease in this special population is multifactorial, resulting both from pre-existing bone metabolism alterations and post-kidney transplant damage, partly due to the use of chronic corticosteroid therapy [40, 41]. A strength of our study is the initiation of denosumab therapy an average of 8 years after surgery, primarily investigating the long-term effects of kidney transplantation on bone metabolism. This may explain the predominance of cortical bone changes in our patients, which appears to contrast with the findings of histomorphometric studies conducted on KTRs that described cortical bone less affected by post-transplant biologic changes in the first years after kidney transplantation [12].

The KDIGO 2017 guidelines recommend DXA for monitoring bone health in CKD patients [42], but it is clear that BMD alone may not fully capture the complex alterations in bone quality that contribute to fracture risk. Bone quality is estimated to account for approximately 30% of bone strength under physiological conditions [11], and this contribution may be even more significant in special populations like KTRs. The contribution of TBS in the CKD population, albeit promising, remains a topic of debate [43]; similarly, the role of 3D-DXA parameters, though promising, also remains under investigation, with some ongoing debate about their clinical utility [44, 45]. Indeed, to the best of our knowledge, 3D-Shaper modeling has not been applied in the KTR setting until now.

In previously published data on aBMD [26], we observed a significant decrease in the Z-score from baseline, indicating a decline in aBMD that is inappropriate for aging and underscoring the deterioration of skeletal health in these patients, a well-documented multifactorial phenomenon [46]. These new findings confirm a similar pattern when bone quality indices are considered. Therefore, in KTRs, the absence of treatment is associated with a marked worsening of bone health, both in terms of quality and quantity. Conversely, denosumab may have the potential to halt or reverse this trend, although in some patients and for certain parameters, its effect might remain insufficient. For this reason, we emphasize the need for a comprehensive approach to bone health and further research into novel osteoactive drugs.

Our data seem to suggest that both TBS and 3D-DXA parameters may provide unique insights into bone quality that are not fully captured by aBMD. We found significant between the investigated parameters and the respective site-specific aBMDs and addressed these concerns by adjusting the tested parameters for the site-specific aBMD; while some parameters did retain significance, further research with larger sample sizes and more robust study designs is needed to confirm these findings.

The limitations of our study, particularly its retrospective non-randomized design and small sample size, therefore the data should therefore be interpreted with caution and further larger confirmatory studies are needed. We emphasize the need for further research to validate these results and better understand the independent role of bone quality parameters like TBS and 3D-DXA in investigating bone microarchitecture and geometry. In addition, adequately powered research is needed to estimate the predictive capability of these tools with regards to fracture risk in this special population.

## Conclusion

In conclusion, four-year long denosumab treatment in a real-life cohort of kidney transplant recipients is associated with significant improvements or stabilization in key bone quality indices, including TBS and 3D-DXA. These findings highlight the potential benefits of denosumab in preserving bone quality in this high-risk population, independently by aBMD changes.

## Supplementary Information

Below is the link to the electronic supplementary material.

Supplementary file1 (JPG 193 KB)

Supplementary file2 (JPG 42 KB)
