# Supplementary figures and images for: Long-Term Changes in Parameters of Bone Quality in Kidney Transplant Recipients Treated with Denosumab
Source: Calcif Tissue Int. 2025 Feb 21;116(1):42. doi: 10.1007/s00223-025-01349-x (PMC11845414; doi:10.1007/s00223-025-01349-x)

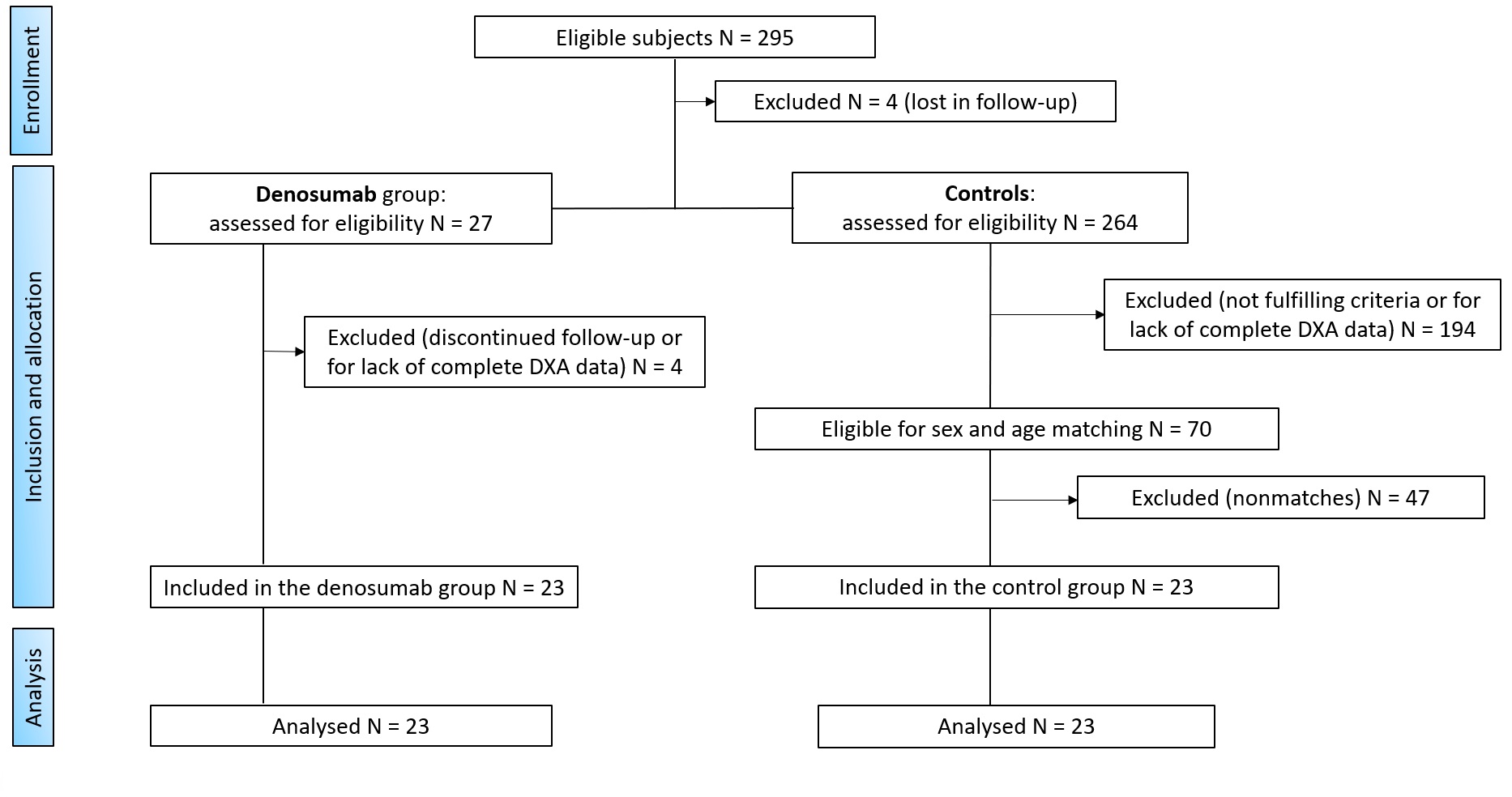

Supplement: Supplementary file 1 — Supplementary file1 (JPG 193 KB) [file 223_2025_1349_MOESM1_ESM.jpg]

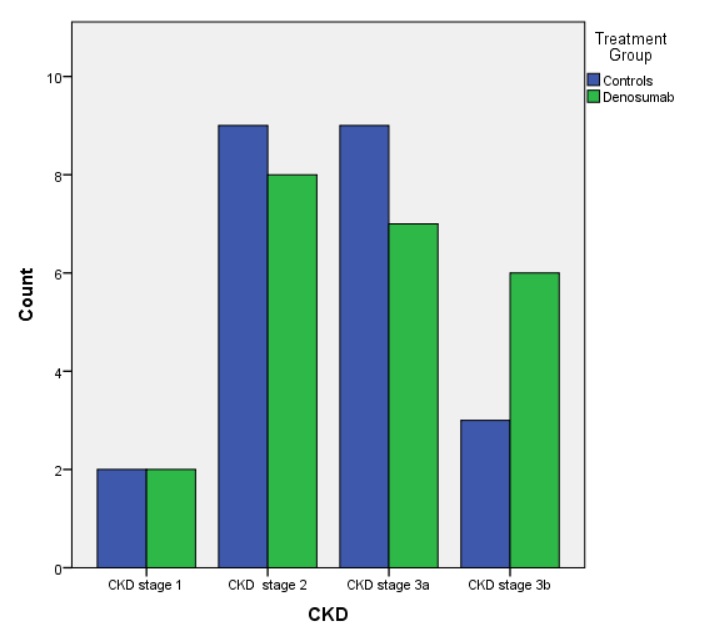

Supplement: Supplementary file 2 — Supplementary file2 (JPG 42 KB) [file 223_2025_1349_MOESM2_ESM.jpg]
